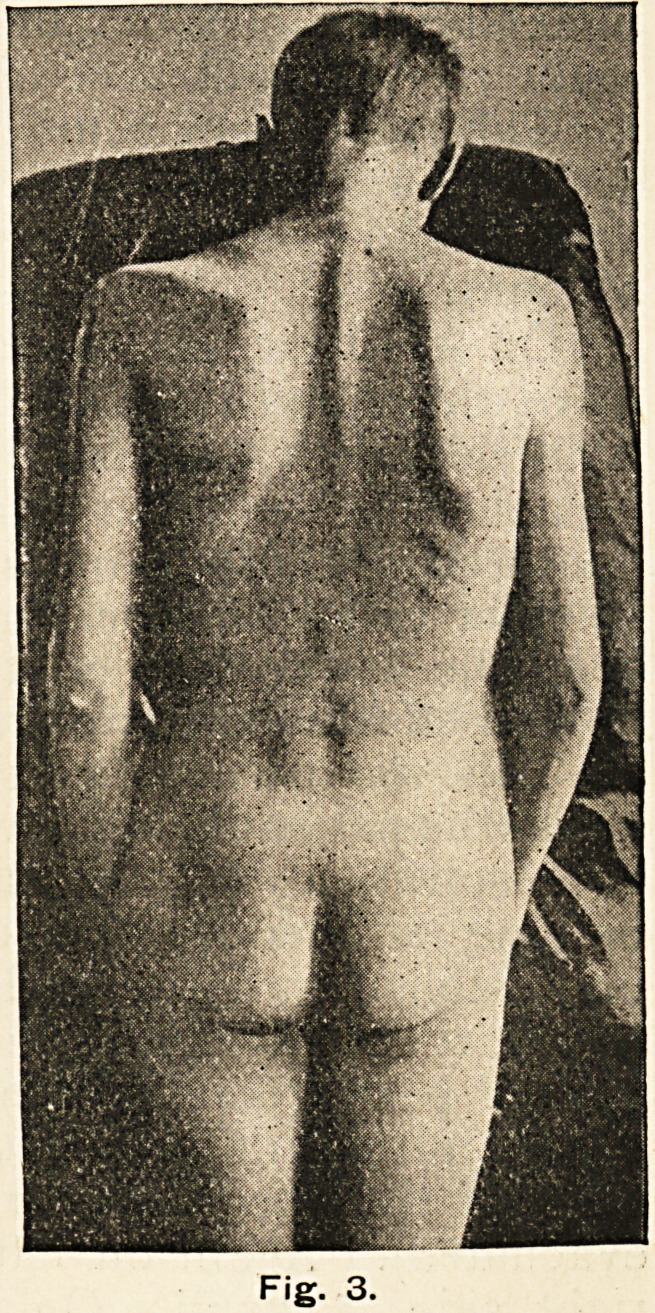# Muscular Atrophy and Sclerodermia

**Published:** 1903-12

**Authors:** J. A. Nixon


### MUSCULAR ATROPHY AND SCLERODERMIA.

BY

J. A. Nixon, M.B., B.C. (Cantab.), M.R.C.P. (Lond.).

The association of muscular atrophy with sclerodermia has been recognised long ago, but originally those observers who noticed this combination of maladies were wont to attribute it to mere coincidence; or, later, as the associated conditions were being more frequently reported, it was customary to explain the atrophy of the muscles as being secondary to the cutaneous changes and directly caused by the compression of the altered skin.

Further research led to the blame being laid upon some ingrowth of connective or fibrous tissue, which, spreading inwards from the skin, bound into bundles the muscle-fibres, interfered with their movement and nourishment, and finally determined their wasting.

Of recent years, however, cases have been recorded, at first isolated, but now somewhat more frequently, in which the changes in the muscles, far from following after the altered state of the skin, precede it, and, indeed, progress without the neighbouring skin being at any time affected.

Stelwagon, in his treatise on diseases of the skin, sums up the observations of other workers in the same field, and shows a large variety of lesions which have been seen with sclerodermia.

Thibierge has found it co-existing with arthritic changes, in which the joints may present the appearance of rheumatoid arthritis, even with ankylosis, a veritable "sclerodactylia"; and similar cases have been reported by Osler, Dercum, Elliott, Uhlenhuth, and others.

Alopecia and leucodermia have been described with sclerodermia, while local trophic disturbances—pain, heat or burning, sense of numbness, diminished sweat, in the affected area—are of frequent occurrence.

With such diverse manifestations, the ætiology of the disease can well be expected to be uncertain and possibly not always constant; yet the cases hitherto collected in which sclerodermia and muscular atrophy are related present so many points of similarity, both in the distribution and course of the affection, that the impression that some single cause governs these changes is wellnigh unavoidable.

The temptation of making all diseases conform to a type is a sufficiently strong one, and possibly many of the greatest errors in medicine have been made owing to this tendency on the part of scientific observers; one likes to pigeon-hole a disease, to minimise the points of contrast between one patient and another, and to bring out into high relief the similarities, so that the final triumph may be achieved of labelling a new disease with a new name, and suggesting a new (and probably "synthetic") preparation as the new remedy. But this is

"fame"—scientific medicine is being compelled to relinquish results for causes; pneumonia is being rapidly "moved on" as an entity to make room for the more comprehensive "pneumococcal infection." And as facts are collected in connection with any single disease, its relations with other diseases are added to, and its connections made use of to arrive at conclusions as to its origin. In this manner, from a consideration of one branch of the conditions allied to sclerodermia, may further light be thrown upon some common cause giving rise to the particular "symptom-complex," and eventually to other possible combinations of symptoms.

One of the most striking features of the disease pointed out by Thibierge in his "Contribution to the Study of Muscular Lesions in Sclerodermia"[1] was the existence of muscular atrophy with healthy overlying skin in subjects who showed the characteristic changes of sclerodermia in other parts of the body.

The cases of generalised sclerodermia, with extensive areas of glossy, thickened skin, robbed of its natural folds, which present the expressionless face and immobile features recognised under the designation of the "masque sclerodermique," are wont to attract attention primarily and often solely to the changes in the skin, while any limitation of movement in the muscles is attributed to the mechanical interference caused by the thickened skin and subcutaneous tissues; but even in these cases the muscle changes have sometimes gone unnoticed until the unusual appearance of the integuments attracted attention, although it may have been that the loss of expression and fixity of the face was in the first place a muscular defect.

The disease has been hitherto looked at too much from the point of view of the dermatologist, and even those authors who have recorded such cases have paid too little attention to those forms in which the muscular changes have occupied parts avoided by the sclerosis of the skin.

A case has, however, recently been in the Royal Infirmary, under the care of Dr. Shaw (by whose courtesy I am enabled to publish these notes), which demonstrated in the most marked

[1] *Rev. de Méd.*, x. 291, 1890.

way the extent to which the muscular atrophy may outrun the changes in the skin.

W. H. C., a man of 35, was admitted to the Infirmary on January 20th of this year. His occupation was that of a postman, with which he was able to combine the trade of a bootmaker. His health had previously been excellent, with this exception, that eight years ago he was confined to bed for a week with "lumbago and pains in the back." He was a single man, and very temperate in his habits.

His parents had ten children, of whom he was the youngest: two of these died from causes unknown, one of his sisters is "asthmatic," the father died in middle age of "congestion of the liver," while his mother is alive and hale; so that, unlike several of the cases of sclerodermia on record, there is no other instance of the affection in the family, neither is there any "neurotic" taint nor predisposition to any "skin" disorders.

The first symptom complained of was a weakness in the limbs, an order of events which I have not been able to find observed in any other cases; at first the left hand felt weak and numb, especially towards night—this was noticed at the outset fifteen months before admission. A month later the right hand followed suit, and aching pains were constantly present in the forearms. Soon after the legs began to ache on walking short distances so much, that after a five-mile round he was obliged to lie down and rest. Finally he was compelled to give up his postman's duties.

Twelve months before he came to the Infirmary the tips of his fingers started tingling, and were so sensitive that he gave up shoemaking, after vainly trying to relieve the tenderness by wearing woollen gloves.

So he at length consulted Mr. Henry, of Minehead, from whom I received an account, saying that in June, 1902, he was complaining of increasing "weakness," that he was very anæmic, listless, and depressed, and also the numbness of the fingers was a prominent symptom.

From July 8th to August 18th the condition was treated with arsenic in moderate doses; but at the end of this time, during which he had taken a complete rest and change of air, some darkening of the skin of the hands and in the flexures of the elbows and knees was detected, and the arsenic was discontinued. None the less, the progressive muscular weakness and the bronzing of the skin persisted and increased to such an extent, that as regards the loss of power the patient could no longer put on his coat, while the pigmentation suggested the possibility of Addison's disease. This was about Christmas, 1902, and in January, as the malady was not stayed, the man came into Bristol.

On admission, the patient presented at first glance a healthy, rather tanned appearance; but the face had a curious fixed, mask-like expression, the skin being yellow, glossy, dry, and giving the idea of being stretched to its utmost in its attempt to cover his face. (Fig. No. 1 conveys something of this condition—the "masque sclerodermique.") The skin here, as in the other parts affected, was of a uniform hue, not mottled; it had lost its supple, elastic feel, did not wrinkle, and, as the patient said, felt "stiff" when he smiled.

Fig. 1.

On the front of the neck and down over the clavicles on to the chest the skin was similarly sclerosed and fixed to the subjacent tissues; over the lower abdomen it became thinner, but the pigmentation was deeper over the pubes and in the fold of the groin.

The skin of the legs was normal, but on the left foot was the scar of an old burn very deeply pigmented. There was no discolouration of the axillæ, while the skin on the upper arms was not thickened and only slightly yellow; on the forearms there was more change, while on the wrists, hands and fingers it was so thick and adherent that it was impossible to pinch it up into the slightest fold. The integument of the back showed only numerous freckles, but no thickening.

Fig. 2.

There were no areas of anæsthesia or hyperæsthesia, the affected skin pitted slightly on pressure, and here and there patches of erythema were present.

Striking as at first sight these cutaneous alterations appeared, the loss of bulk in the muscles was even more noteworthy. The shoulder girdle suffered to the greatest extent, and the natural poise of the head drew particular attention to the wasting of the muscles at the back of the neck, for the head was carried in an attitude expressive of deep humility or profound meditation. (Fig. 2.)

The right side was more advanced in atrophy than the left, and the chief muscles involved were the deltoid, trapezius, supraspinatus, rhomboids, teres and triceps; each biceps was represented by a cord not thicker than a man's forefinger, and all the forearm muscles were much diminished in size.

The pectorals and latissimus dorsi, the anterior muscles of the neck and the legs were little if at all altered; but the glutei seemed gone, and the nates were composed almost entirely of fat. (Fig. 3.)

Fig. 3.

Not only were the movements of the limbs impaired by reason of the muscular atrophy, but the condition of the skin interfered with certain of them. The arms could not be raised above the head, as the skin of the front of the chest became tense and unyielding; the fingers for the same cause could not be completely extended. Mastication of food was a tedious process, and deglutition was slow and difficult.

The legs so far had escaped, but the calves felt "stiff." However, the patient could walk well and naturally, complaining only of being easily tired; if laid on the floor, he could not get up without help, owing probably to the weakness of the glutei. His method of rising from a lying position was precisely that of a child suffering from *pseudo-hypertrophic paralysis*: that is to say, he rolled on to his face, and, raising himself on to his knees, "climbed up his legs" with the utmost difficulty and unsteadiness.

Electrical reactions.

*Faradic current.*—No response in supra- and infra-spinati, trapezius, or muscles of upper arm.

Diminished.—Teres and rhomboidei: muscles of thigh and buttock. Normal in muscles of arms and legs, the peronei responding the most readily.

*Galvanic current.*—Reaction of degeneration in teres and thigh muscles. No response in supra- and infra-spinati, trapezius, deltoids.

The general health of the patient was good, except for the fact that he was easily tired. Temperature normal. Pulse 80: small volume, low tension, and regular. The heart's apex beat was in the fifth space just inside the nipple-line, and the only noticeable defect was a feebleness of the first sound. The lungs appeared healthy in every respect. Urine 1030; no albumin, no sugar.

Fundus oculi, normal in every detail. No trace of a thyroid gland could be felt.

*Reflexes.*—Knee jerks very sluggish. Plantar reflex with difficulty obtained, but flexor type. No ankle clonus. Pupils react to light and accommodation.

The subsequent course of the disease was complicated by a series of untoward events, and the foregoing description of the cardiac and respiratory systems is given somewhat fully on this account.

On April 1st, after being in the Infirmary for two months or more, there was a sudden development of pleural friction on the right side, with fever ranging between 100° and 102°; the pulse became very feeble and irregular. There was a tendency to faintness, the heart dropping an occasional beat, and a murmur, whether endo- or exocardial it was difficult to say, was heard over the lower end of the sternum. Subsequently, double pleural effusion appeared, and with it loud pericardial friction.

This was no mere passive complication, but for four or five weeks the patient hung between life and death; he wasted rapidly, the respiration was grievously embarrassed, and the heart-failure became so serious a symptom that his recovery appeared impossible: on many nights the pulse was imperceptible and the heart sounds were inaudible. There was no evidence at any time of pericardial effusion, though the friction persisted.

Aspiration of the pleural cavity was performed on both sides of the chest on April 21st (R. Oij.ss. L. Oj. clear fluid evacuated). Again on left side April 27th, ℥xxxv. May 6th, right side, ℥xxxvij. On each occasion the fluid was serous, sterile, and no tubercle bacilli were found.

Shortly after the onset of these threatening complications the right arm became flexed at an angle of nearly 90° at the elbow, and the biceps could be felt as a tense cord, no thicker than the little finger, lying under normal, elastic skin, which could be easily raised from the underlying muscle; a lesser degree of contraction was later manifested in the left biceps, whose bulk was similarly diminished.

Contrary to all expectation, the patient lived on from hour to hour, almost pulseless, breathing at varying intervals a shallow breath that bade fair to be the last, and by night on several occasions the sister of the ward was told that art could do no more. He did none the less hold his own, aided by strychnine and brandy to a trifling extent; but of all the stimulants resorted to, none could be found to approach tincture of musk in drachm doses for rapidity and certainty of effect.

It was not until the middle of May that any real improvement was noted; then, as inexplicably as it had come on, the signs of heart failure retreated, the pleuræ no longer filled with fluid, and the flagging strength revived.

On June 26th, for the first time since April 1st, the patient was lifted out of bed on to a couch. A month later he could stand with help, and on September 12th he was able to be moved to his home in Minehead, strong enough to walk across a room.

His lungs had cleared up, save from some dulness in the right axilla and scanty crepitations. The heart sounds were feeble and the apex not located, but the cardiac dulness was not increased.

He had no dropsy, though this had been universal when the heart was at its worst, and the breathing was full and easy.

As for his muscular system, so general was the wasting, that it was difficult to allot the probable shares to the primary affection or the intercurrent disease.

The sclerosis of the skin of the forearms and hands had progressed, and also of the upper part of the chest. The skin of the upper arms was still natural and thin, yet the contracture of the biceps had increased remarkably, the right arm being incapable of extension to much more than a right angle, while the muscles felt like mere cords in each arm. The muscles of the shoulder girdle were more wasted than on admission, and the legs were reduced to sticks; yet none of the thigh or calf muscles had contracted like the biceps.

Although repeated examinations of the pleural fluid failed to reveal the tubercle bacilli, and there was never any sputum to examine, it was difficult to put aside the idea of a tubercular pleurisy, for the pleurisy was sufficiently in advance of the heart failure to preclude the theory of a passive hydrothorax; and during the early days of the patient's stay in the ward the adjoining bed had been occupied by a man with phthisis, whose lungs were riddled with cavities and his sputum copious and purulent, teeming with tubercle bacilli.

Here then was a case in which the changes in the skin did not bear any causal relationship to the atrophy of the muscles: in the majority of the muscles which had degenerated there was no mechanical restraint exercised upon their movements by sclerosed skin and subcutaneous tissues; there was no ingrowth of fibrous tissue spreading down from the deeper layers of the integument, for in many places the skin was thin and elastic, and could be without difficulty raised, as in the case of the biceps, from the muscle beneath.

The semiflexion of the arms was a prominent feature, but it does not appear to have been an instance of the joints becoming fixed "by the contracted integument" (*vide* Abraham in Allbutt's *System*).

M'Guire was one of the first to describe a case in which the atrophy outran the sclerodermia, and Thibierge collected accounts of five cases—by himself (1), Westphal (2), Goldschmidt and Schultze—with autopsies; but the investigations were not sufficiently complete, as Thibierge himself admits, to throw much light on the ætiology of the disease, especially with reference to the condition of the central nervous system.

Sclerodermia is not infrequently found in conjunction with symptoms of Graves' disease (Singer, Jeanselmes, Ditscheim, Grünfeld, Osler, Uhlenhuth and others), with the phenomena of Raynaud's disease (Hutchinson, White), and with other manifestations of abnormal pigmentation or leucoderma. The thyroid was noticeably atrophied in this case. The early symptoms are often local pain of a neuralgic or rheumatic type, sensations of numbness, and joint symptoms may coexist, all of which are frequent accompaniments of central nerve lesions, so that the view has been adopted by some that the cause is a neurosis—an angeio- or tropho-neurosis; and although many investigators have failed to discover any pathogenetic changes in the central nervous system, Westphal and Jacquet have described cord lesions.

The muscular atrophy in this case was not only consistent with a central cause, but could scarcely from its distribution and progress be associated with any other (including, of course, such generalised lesions as peripheral neuritis).

Many local phenomena of disease of the central nervous system are known, and identical lesions may have varying localisations; it is to some such group, which already includes trophic changes in the skin and joints, that this form of muscular atrophy may have for the present to be added.

The exact nature and causes are far from being determined; but, as Sir James Paget said of "rare and new diseases," that the cases collected even as they are, singly and in disorder, need not be set aside with idle thoughts or idle words about "curiosities" or "chances." Not one of them is without a meaning, not one but might be the beginning of excellent knowledge, if only we could answer the question, "Why is this rare? or, being rare, Why did it in this instance happen?"

## Figures and Tables

**Fig. 1. f1:**
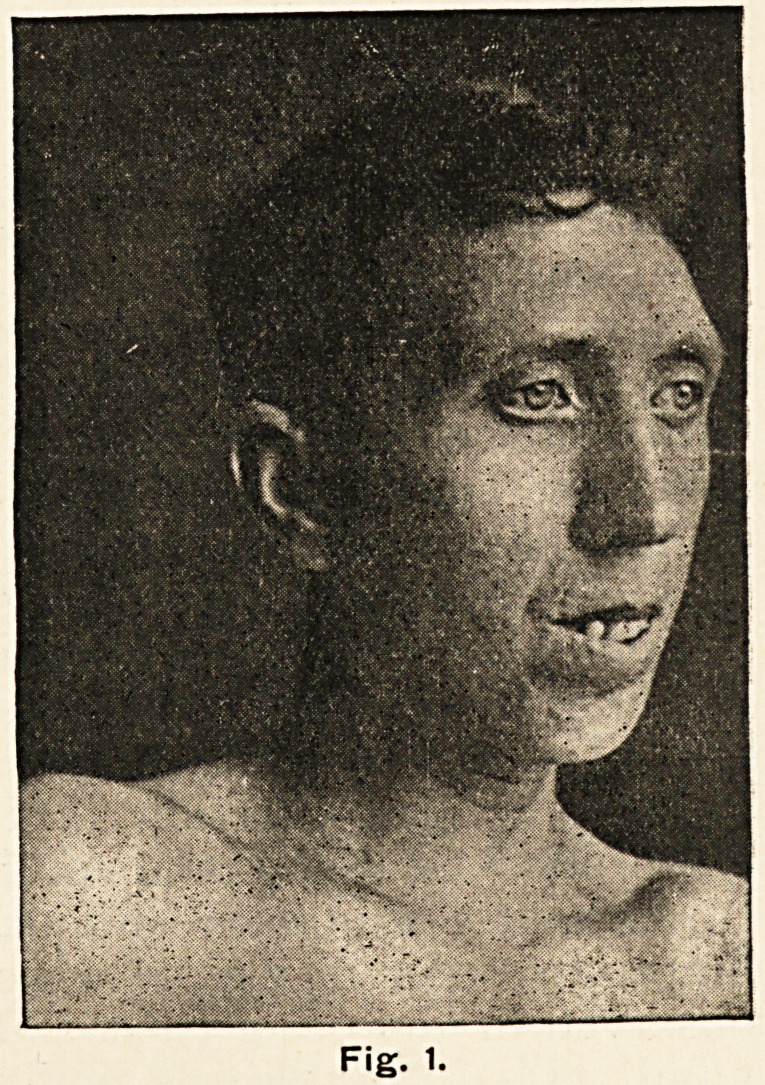


**Fig. 2. f2:**
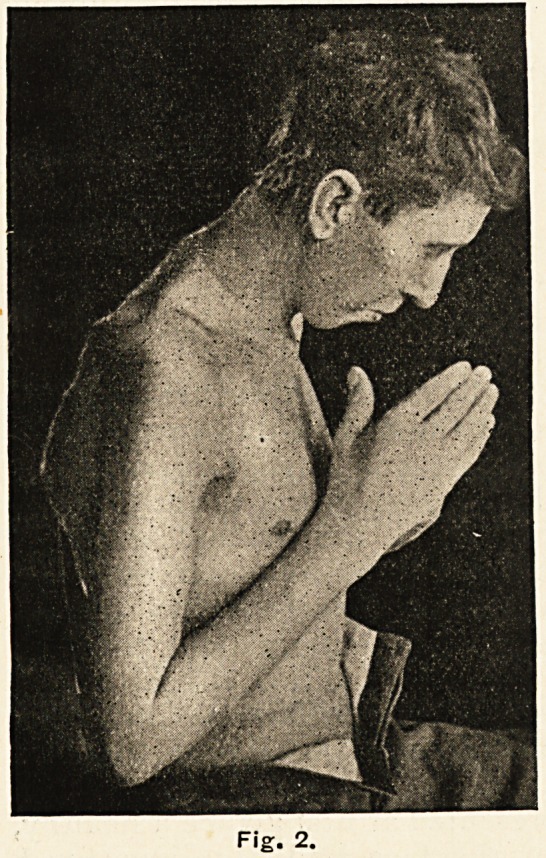


**Fig. 3. f3:**